# Automated multiple trajectory planning algorithm for the placement of stereo-electroencephalography (SEEG) electrodes in epilepsy treatment

**DOI:** 10.1007/s11548-016-1452-x

**Published:** 2016-07-01

**Authors:** Rachel Sparks, Gergely Zombori, Roman Rodionov, Mark Nowell, Sjoerd B. Vos, Maria A. Zuluaga, Beate Diehl, Tim Wehner, Anna Miserocchi, Andrew W. McEvoy, John S. Duncan, Sebastien Ourselin

**Affiliations:** 1 Centre for Medical Image Computing, University College London, London, UK; 2 Department of Clinical and Experimental Epilepsy, UCL Institute of Neurology, London, UK; 3 National Hospital for Neurology and Neurosurgery (NHNN), London, UK; 4 Dementia Research Centre, Department of Neurodegenerative Disease, UCL Institute of Neurology, London, UK

**Keywords:** Computer-assisted planning, Epilepsy, Neurosurgery, Image-guided neurosurgery

## Abstract

**Purpose:**

About one-third of individuals with focal epilepsy continue to have seizures despite optimal medical management. These patients are potentially curable with neurosurgery if the epileptogenic zone (EZ) can be identified and resected. Stereo-electroencephalography (SEEG) to record epileptic activity with intracranial depth electrodes may be required to identify the EZ. Each SEEG electrode trajectory, the path between the entry on the skull and the cerebral target, must be planned carefully to avoid trauma to blood vessels and conflicts between electrodes. In current clinical practice trajectories are determined manually, typically taking 2–3 h per patient (15 min per electrode). Manual planning (MP) aims to achieve an implantation plan with good coverage of the putative EZ, an optimal spatial resolution, and 3D distribution of electrodes. Computer-assisted planning tools can reduce planning time by quantifying trajectory suitability.

**Methods:**

We present an automated multiple trajectory planning (MTP) algorithm to compute implantation plans. MTP uses dynamic programming to determine a set of plans. From this set a depth-first search algorithm finds a suitable plan. We compared our MTP algorithm to (a) MP and (b) an automated single trajectory planning (STP) algorithm on 18 patient plans containing 165 electrodes.

**Results:**

MTP changed all 165 trajectories compared to MP. Changes resulted in lower risk (122), increased grey matter sampling (99), shorter length (92), and surgically preferred entry angles (113). MTP changed 42 % (69/165) trajectories compared to STP. Every plan had between 1 to 8 (median 3.5) trajectories changed to resolve electrode conflicts, resulting in surgically preferred plans.

**Conclusion:**

MTP is computationally efficient, determining implantation plans containing 7–12 electrodes within 1 min, compared to 2–3 h for MP.

## Introduction

Between 20 and 40 % of focal epilepsy patients are refractory to antiepileptic medications [13]. Such patients are candidates for curative surgery, which aims to resect the epileptogenic zone (EZ) that generates seizures [3]. In about 25 % of surgical candidates, the EZ cannot be inferred from noninvasive imaging data, and intracranial electroencephalography (EEG) is needed to identify the EZ [7].

Stereo-EEG (SEEG) records EEG signals via depth electrodes surgically implanted in the brain. SEEG electrodes record from a 1-cm core around the cerebral entry to the distal end (target) that may be placed in hippocampus, amygdala, or midline or neo-cortex in temporal, frontal, parietal, or occipital lobes. Electrode implantation carries a risk of haemorrhage, neurologic deficit, and infection [4].

Preoperative planning of electrode trajectories, defined by the target and the skull entry point, can minimise implantation risk by ensuring electrodes avoid critical structures (e.g. arteries, veins, sulci) and conflicts between electrodes. Planning may also improve the efficiency of the SEEG recording by ensuring electrodes pass through the maximal amount of grey matter (GM), GM being the component of brain tissue that generates seizures. Current clinical practice for planning electrode trajectories involves manual evaluation of trajectories in series. This is a complex, time-consuming task requiring:

1. Integrating information across imaging modalities to locate critical structures, GM, and targets.
2. Optimising several criteria for each trajectory to sample the target, avoid critical structures, and obtain a suitable angle to traverse the skull.
3. Adjusting trajectories to maximise GM capture and avoid electrode conflicts, whereby two electrodes may contact each other. Placing a new electrode may require adjusting previously planned trajectories.

Computer-assisted planning algorithms can reduce planning time by calculating quantitative measures of trajectory suitability. These measures can be used to select the best trajectory (automated planning) or inform manual trajectory selection (assisted planning).

We present an automated multiple trajectory planning (MTP) algorithm that calculates a combination of trajectories, or plan, for a set of targets. Trajectories are assessed on proximity to critical structures (risk score) and sampling of GM [GM-white matter (GM-WM) ratio]. Our MTP algorithm uses dynamic programming to reduce the search space and a depth-first search to find a plan whereby each electrode trajectory is surgically feasible, does not interfere with other trajectories, avoids critical structures, and maximises GM sampling. MTP is integrated into the EpiNav$^{\mathrm{TM}}$ software platform [29] to enable manual trajectory assessment.

The remainder of the manuscript is organised as follows. The third section describes the previous work in computer-assisted trajectory planning. The fourth section describes our MTP algorithm. The fifth section describes the evaluation of our MTP algorithm. The sixth section discusses MTP, and the seventh section provides concluding remarks.

## Previous work in trajectory planning

Trajectory planning algorithms have been developed for deep brain stimulation (DBS) electrodes [1, 2, 8, 14], biopsy needles [11, 16, 20, 23, 24], or SEEG electrodes [5, 6, 26, 27, 29]. These methods provide either: (1) assisted planning to aid manual trajectory selection [11, 16, 20]; (2) automated planning for a single trajectory planning [1, 2, 8, 10, 14, 24, 25, 29]; or (3) automated multiple trajectory planning [5, 6, 26, 27].

Assisted planning methods aim to reduce the time and complexity of manual trajectory selection by displaying measures of risk for potential trajectories [11, 16, 20, 22]. [16] displayed a heat map corresponding to the minimum distance to critical structures for potential entry points. Similarly, [11] displayed an entry point safety map, safety being related to distance from critical structures. [20] reduced computation time for safety maps using graphical processing units (GPUs) to enable real-time user interaction. [22] displayed a cumulative risk, the summation of distance from critical structures along the trajectory.

**Fig. 1:**
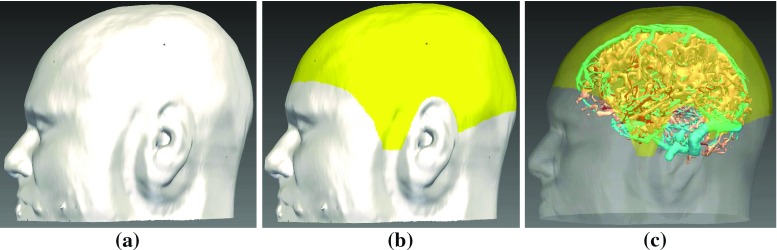
Example study displaying **a** skull segmentation (*white*), **b** skull template (*yellow*) that excludes surgically infeasible regions, and **c** veins (*cyan*), arteries (*red*), and sulci (*peach*) with skull (semi-transparent *white*) and skull template (semi-transparent *yellow*)

Single trajectory planning algorithms automatically determine the best trajectory for one electrode given a specific quantitative measure. [25] assessed trajectories using a risk score calculated by summing traversal costs, where regions to be avoided had a high traversal cost, along the trajectory. Similarly, [10] summed traversal costs along the trajectory but added a penalty for trajectories near blood vessels to reduce the risk of haemorrhage. [1] assessed trajectories by first removing potential trajectories that were an unsafe distance from critical structures. The remaining trajectories were assessed by a weighted sum of (1) the minimum distance to critical structures and (2) the cumulative distance from all critical structures. [23] calculated a traversal cost by first computing a per pixel risk score, based on distance to critical structures. They then determined two risk scores: (1) maximum risk along the trajectory and (2) a summation of the risk along the trajectory. The user could select which of these two risk scores to use. [8] developed a generic optimisation algorithm for trajectory planning, allowing a user to define a set of hard constraints, rules that must not be violated, and soft constraints, rules that could be minimised. The generic optimisation eliminates trajectories that violate the hard constraints and then finds the trajectory which minimises the summation of soft constraints. [2] used a similar approach, defining hard constraints, specific entry points and avoiding critical structures (ventricles, blood vessels, sulci), and soft constraints, minimising overlap with the caudate and GM. [14] combined 6 soft constraints and 2 hard constraints to define a weighted cost function that determined the best trajectory for targeting the subthalamic nucleus. [26] developed an algorithm that maximises distances from critical structures and GM sampling. [24] developed an algorithm that optimises a weighted sum of the trajectory distance to critical structures so that blood vessels, with a high weight, are always avoided, while WM tracts and regions of cortical function, with a low weight, may be traversed if no other path exists. Constraints in [24] are specific to placing electrode for DBS and may not be generalisable to SEEG electrode implantation.

Multiple trajectory planning algorithms determine the best combination of trajectories, or plan, for multiple electrodes. Multiple trajectory planning not only takes into account the quantitative measures for individual electrodes but also that electrodes must not contact each other. [26] optimised three electrodes with targets in the amygdala, anterior and posterior hippocampus so that risk was minimised and there were no conflicts between electrodes. In [26] potential entry points were constrained by the use of target specific entry map priors. A more extensive evaluation of this method on 37 patients was presented in [27] in which increased GM sampling by optimising the number of electrode contacts in GM was demonstrated.

[5] presented a multiple trajectory planning algorithm that operates serially. The algorithm sets the first electrode to the best trajectory, and additional electrodes are set by removing trajectories that interfere with existing electrodes, and then selecting the best trajectory. This algorithm is dependent on trajectory order and may not find an optimal plan. [6] overcame these limitations by evaluating all potential plans and returning the best plan. However, enumerating all potential plans is computationally expensive when evaluating many targets or many potential trajectories per target. To reduce the number of potential plans, [6] reduced potential entry points by randomly sampling within a user-specified region.

Previous work from our group presented an automated entry point search algorithm [29] in which all points on the skull were potential entry points, removing the need to manually specify entry regions or define entry map priors for each target. All points on the skull result in 2000–10,000 potential entry points, and for eight targets this corresponds to a minimum of $1\text {E}26$ potential plans.

We present a MTP algorithm that combines dynamic programming to reduce the number of potential plans, and depth-first searching, to find a suitable plan. Trajectories are assessed by risk score, measured as the cumulative distance to blood vessels from the trajectory [29], and GM sampling, measured as the proportion of electrode contacts that are in GM. Our algorithm is an improvement over the current state of the art in that it: (1) finds a combination of electrode trajectories with no limitations on the number or order of electrodes and (2) is computationally efficient, finding a plan with 7 to 12 electrode in under one minute, and (3) is integrated in the EpiNav$^{\mathrm{TM}}$ software platform to enable manual assessment.

## Multiple trajectory planning algorithm

A trajectory is defined as $v = \lbrace T, E, R, G \rbrace$ where *T* is the target in the brain, *E* is the entry on the skull, *R* is the risk score, and *G* is the GM-WM ratio. For a set of *N* targets a plan is defined as $V( N ) = \lbrace v_{1,a_{1}},\ldots , v_{N,a_{N}} \rbrace : a_{i} \in \lbrace 1,\ldots , M_{i} \rbrace , i \in \lbrace 1, \ldots , N \rbrace$ where $M_{i}$ is the number of potential trajectories for the $i^{\mathrm{th}}$ target. The plan *V*( *N* ) is defined such that each trajectory attains one of *N* targets. MTP finds a plan $V_{\mathrm{min}}( N )$ that attains all targets, minimises *R*, maximises *G*, and avoids conflicts between electrodes.

Prior to trajectory planning, segmentation of the skull and critical structures is performed as described in the section “Critical structure extraction”. For each target $T_{i}$, a risk score $R_{i,a_{i}}$, that quantifies proximity to blood vessels, and a GM-WM ratio $G_{i,a_{i}}$, that quantifies GM sampling, are calculated as described in the section “Single trajectory planning algorithm”. The MTP algorithm described in the section “Multiple trajectory planning algorithm” calculates $V_{ \mathrm{min}}( N )$. The EpiNav$^{\mathrm{TM}}$ software platform enables manual assessment of $V_{\mathrm{min}}( N )$ as described in the section “Plan visualisation and assessment”.

### Critical structure extraction

Automated trajectory planning is dependent on accurately segmenting critical structures (arteries, veins, and sulci), GM, and the skull surface. Algorithms chosen for these tasks are currently being used in the clinic to generate 3*D* models for manual trajectory planning and have been used in presurgical patient evaluation for over 4 years [19, 21]. Blood vessels are segmented with a customised vessel extraction tool [30] from CT angiography, 3*D* phase contrast MRI, or T1-weighted MRI with gadolinium enhancement. GM and the cortex were segmented using FreeSurfer [9]. Sulci were extracted from the cortex surface. Figure 1c illustrates an example segmentation for veins (cyan), arteries (red), and sulci (peach).

Skull segmentation with template registration constrains entry points to regions suitable for implantation. A patient-specific skull is segmented from a CT scan using thresholding and morphologic dilation to ensure a fully connected surface. The template skull is aligned to the patient skull using Iterative Closest Points (ICP) [28] to minimise the distance between the two surfaces. The template skull excludes regions inappropriate for implantation such as the face, ears, and regions inferior to the transverse sinus. Figure 1 shows an example patient (white) and template (yellow) skull.

### Single trajectory planning algorithm

Previous work from our group [29] presented real-time automated single trajectory planning (STP) for a target $T_i$. STP calculates potential entry points $\hat{E}_{i,a_{i}} : a_{i} \in \lbrace 1, \ldots , M_{i} \rbrace$ by considering trajectory length and entry angle (described in the section “Entry point search”). Next trajectories, defined as $\overline{\hat{E}_{i,a_{i}} T_{i}}$, that intersect critical structures (arteries, veins, or sulci) are removed from consideration. Then at evenly spaced points along each trajectory $x \in \overline{E_{i,a_{i}} T_{i}}$ the distance to the nearest blood vessel $f_{\mathrm{crit}}(x)$ is found (described the section “Bounding volume hierarchy (BVH) for trajectory evaluation”). Finally for $\overline{E_{i,a_{i}} T_{i}}$ a risk score $R_{i,a_{i}}$, computed from $f_{\mathrm{crit}}(x)$, and a GM-WM ratio $G_{i,a_{i}}$ are calculated (described in the section “Trajectory ranking”). A stratified ranking algorithm sorts trajectories by first minimising $R_{i,a_{i}}$ and then maximising $G_{i,a_{i}}$.

#### Entry point search

Potential entry points $\hat{E}_{i,a_{i}} : a_{i} \in \lbrace 1, \ldots , M_{i} \rbrace$ are identified by considering all vertices in the template skull mesh. $\hat{E}_{i,a_{i}}$ are removed from consideration based on the following criteria:

1. **Trajectory length** The length of $\overline{\hat{E}_{i,a_{i}} T_{i}}$ must be shorter than $d_{{\mathrm{length}}}$, the maximum electrode length.
2. **Entry angle** The angle between $\overline{\hat{E}_{i,a_{i}} T_{i}}$ and the skull normal must be less than $d_{{\mathrm{angle}}}$, the angle that can be accurately drilled.

Calculating these exclusion criteria for $\overline{\hat{E}_{i,a_{i}} T_{i}}$ is computationally inexpensive; hence, it is practical to remove $\hat{E}_{i,a_{i}}$ that do not meet these criteria first.

#### Bounding volume hierarchy (BVH) for trajectory evaluation

Each trajectory $\overline{\hat{E}_{i,a_{i}} T_{i}}$ is tested for intersection with critical structures (arteries, veins, sulci) using a bounding volume hierarchy (BVH) to enable real-time calculation. Trajectories that intersect these structures are removed from consideration. All remaining trajectories are sampled at 128 evenly spaced points *x* such that $x\in \overline{E_{i,a_{i}} T_{i}}$. For every *x*, the distance to the nearest blood vessel (arteries, veins) $f_{\mathrm{crit}}(x)$ is calculated. BVH construction and traversal are described below.

*Bounding volume hierarchy construction* For each critical structure (arteries, veins, and sulci) a BVH is constructed as in [12]. Each triangle in the surface is assigned a 30-bit Morton code [15], calculated by combining the 10-bit Morton code of each triangle vertex coordinate. An efficient bit-wise sorting of the triangles is performed using the Morton codes. The BVH is created by iteratively splitting triangles according to the highest different bit between Morton codes. This is repeated until each leaf node contains one triangle. Finally, for every node a bounding box is calculated. For each leaf node the bounding box is calculated as the smallest rectangle that contains the triangle. The bounding box for all other nodes is the union of the bounding boxes of their children nodes.

*Bounding volume hierarchy traversal* BVH traversal detects collision of $\overline{\hat{E}_{i,a_{i}} T_{i}}$ with each critical structure. Initially the top BVH node is added to the queue. If $\overline{\hat{E}_{i,a_{i}} T_{i}}$ intersects the bounding box of the first node in the queue, its children nodes are added to the queue. Once a leaf node is reached $\overline{\hat{E}_{i,a_{i}} T_{i}}$ is removed from consideration if it intersects the triangle of the leaf node.

For the remaining trajectories, the closest distance between each point $x \in \overline{E_{i,a_{i}} T_{i}}$ and the $j\hbox {th}$ critical structure $f_{j}(x)$ is calculated. For this computation sulci are not included. Initially the top BVH node is added to the queue and $f_{j}(x) = \infty$. The first node in the queue is removed, and the distance between each child node and its bounding box $f_{bb}(x)$ is calculated. For a point inside the bounding box $f_{bb}(x) = 0$. If $f_{bb}(x) < f_{j}(x)$ the node is added to the queue so that the first node corresponds to the smallest value of $f_{bb}(x)$. For a leaf node the distance between *x* and the triangle is computed, if $f_{tri}(x) < f_{j}(x)$, then $f_{j}(x) = f_{tri}(x)$. This is repeated until no nodes are in the queue. After all critical structures have been evaluated, the closest distance is calculated as $\displaystyle f_{\mathrm{crit}}(x) = {{\mathrm{arg\,min}}}_{j} ( f_{j}(x))$.

#### Trajectory ranking

Entry points that meet all hard constraints, $E_{i,a_{i}}: a_{i} \in \lbrace 1, \ldots , M_{i} \rbrace$, are ranked by risk score $R_{i,a_{i}}$, a measure of cumulative distance from blood vessels, and GM-WM ratio $G_{i,a_{i}}$, a measure of GM capture.

**Fig. 2:**
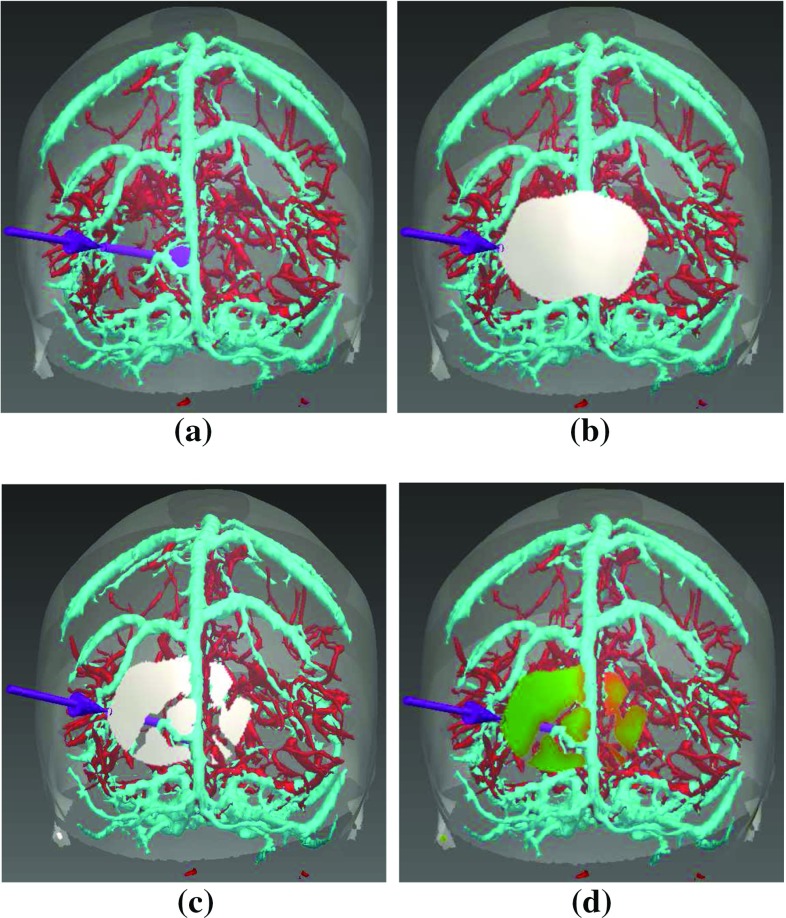
Entry point search algorithm. Critical structures are arteries (*red*), veins (*cyan*), and sulci (not shown). The lowest risk trajectory is displayed in purple (*arrow* indicating entry and *sphere* indicating target). **a** Initial set of potential entry points $\hat{E}_{i,a_{i}}$ obtained from the skull template (semi-transparent *white*). **b** Opaque white patch corresponds to $\hat{E}_{i,a_{i}}$ after excluding trajectories due to length or angle. **c** Opaque white patch corresponds to $\hat{E}_{i,a_{i}}$ after excluding trajectories due to critical structure intersection. **d** Coloured patch corresponds to risk scores $R_{i,a_{i}}$ with low (0—*green*) to high (1—*red*) risk

*Risk score* The risk score $R_{i,a_{i}}$ measures cumulative distance to blood vessels. The trajectory $\overline{E_{i,a_{i}} T_{i}}$ has a high risk if the nearest critical structure is less than a “Safety Margin”, determined by the user-defined value $d_{\mathrm{safety}}$. If $\overline{E_{i,a_{i}} T_{i}}$ has a distance to the nearest critical structure greater than a “Risk Zone”, determined by the user-defined value $d_{\mathrm{risk}}$, it has no potential risk.

The cumulative distance of risk along $\overline{E_{i,a_{i}} T_{i}}$ is calculated as,

1 $$\begin{aligned} S_{{\mathrm{crit}}} = \int _{E_{i,a_{i}}}^{T_{i}} d_{{\mathrm{risk}}} - ( f_{{\mathrm{crit}}}(x)-d_{{\mathrm{safety}}} ) dx, \end{aligned}$$

where $f_{{\mathrm{crit}}}(x)$ is the distance between *x* and the nearest critical structure. For normalisation purposes if $f_{{\mathrm{crit}}}(x) > d_{{\mathrm{risk}}}$, then $f_{{\mathrm{crit}}}(x)= d_{{\mathrm{risk}}}$ so that the final value the *x* contributes to the risk score is zero. If $f_{{\mathrm{crit}}}(x) < d_{{\mathrm{safety}}}$, then automatically $R_{i,a_{i}} = 1$, representing the highest risk.

The final risk $R_{i,a_{i}}$ is normalised to the range [0, 1], where 0 corresponds to no risk and 1 corresponds to the highest risk. $R_{i,a_{i}}$ is calculated as,

2 $$\begin{aligned} R_{i,a_{i}} = \dfrac{S_{{\mathrm{crit}}}}{ (d_{{\mathrm{risk}}}-d_{{\mathrm{safety}}}) * { length}}, \end{aligned}$$

where ${ length}$ is the length of $\overline{E_{i,a_{i}} T_{i}}$. Figure 2d displays $R_{i,a_{i}}$ as a heat map from low (0-green) to high (1-red) risk.

*Grey matter-white matter ratio* GM-WM ratio measures the proportion of electrode contacts in GM. GM-WM ratio corresponds to the SEEG efficiency for each trajectory as GM generates seizures. For each trajectory $\overline{E_{i,a_{i}} T_{i}}$ a set of *J* contact points, $c_{j} : j \in \{1, \ldots , J \}$ each with a sampling radius $c_r$ are defined. Each contact point is assessed if $c_{j} \pm c_{r}$ is located in GM. GM-WM ratio is calculated as,

3 $$\begin{aligned}&G_{i,a_{i}} \nonumber \\&\quad = \dfrac{ \sum _{j = 1}^{J} (H[f_{gm}(c_j - c_r) ] + H[f_{gm}(c_j) ] +H[ f_{gm}(c_j + c_r) ] }{3 * J}, \nonumber \\ \end{aligned}$$

where $f_{gm}(\cdot )$ is the signed distance at $c_j$ from the GM surface, $H[\cdot ]$ is the Heaviside function, and *J* is the number of contact points. $H[\cdot ]$ is defined so negative values, locations inside GM, are 1 and positive values, locations outside GM, are 0. Similar to $f_{{\mathrm{crit}}}(x)$ a BVH is used to calculate $f_{gm}(\cdot )$.

*Stratified ranking* Trajectories are first ranked by $R_{i,a_{i}}$ so that $v_{i,1}$ has the lowest risk. Next trajectories are placed into *K* histogram bins so the $k^{\mathrm{th}}$ bin contains $v_{i,a_i}: (k-1)/K \le a_i < k/K$. Within each bin trajectories are ranked according to $G_{i,a_{i}}$ so $v_{i,1}$ has the highest GM-WM ratio.

### Multiple trajectory planning algorithm

MTP aims to find the best plan $V_{{\mathrm{min}}}( N ) = [ v_{1,a_{1}}, \ldots , v_{N,a_{N}}] : a_{i} \in \{1, \ldots , M_{i}\}, i \in \{1, \ldots , N \}$ with no electrode conflict. Electrode conflict occurs when two trajectories are closer than a user-defined value $d_{{\mathrm{traj}}}$. $V_{{\mathrm{min}}}( N )$ is defined as,

4 $$\begin{aligned} R_{{\mathrm{total}}}&= {{\mathrm{arg\,min}}}_{V_{{\mathrm{min}}}( N )} \bigg ( \dfrac{1}{N} \sum _{i=1}^{N} R_{i,a_{i}} \bigg ) \nonumber \\&\text { s.t. } D(\overline{E_{i,a_{i}} T_i}, \overline{E_{j,a_{j}} T_j} ) > d_{{\mathrm{traj}}}: \forall i,\nonumber \\ {}&\quad \ \forall j \in \{ 1, \ldots , N\}, i \ne j, \end{aligned}$$

where $D( \cdot , \cdot )$ is the minimum Euclidean distance between two line segments. A depth-first search with dynamic programming to limit potential plans is used to calculate $V_{{\mathrm{min}}}( N )$ as described in the sections “Depth-first search algorithm” and “Dynamic programming for determining potential combinations”.

#### Depth-first search algorithm


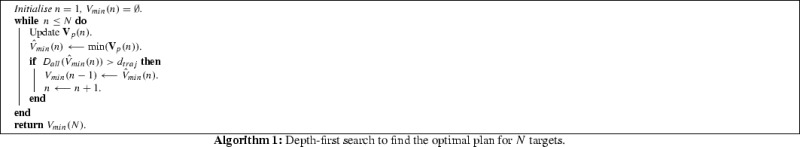


Algorithm 1 iteratively (1) calculates $\hat{V}_{{\mathrm{min}}}(n)$, a suitable plan for *n* trajectories and (2) rejects $\hat{V}_{{\mathrm{min}}}(n)$ if electrodes conflict. At each iteration of Algorithm 1 a set of low risk plans, defined as ${\mathbf {V}}_{p} (n) = [V_1(n), \ldots , V_q(n)]$, is calculated. For the lowest risk plan $\hat{V}_{{\mathrm{min}}}(n) \in {\mathbf {V}}_{p} (n)$ the function $D_{{\mathrm{all}}}( \cdot )$ detects conflict between electrodes by finding the minimum Euclidean distance between all pairs of trajectories in $\hat{V}_{{\mathrm{min}}}(n)$ (i.e. $\min (D(\overline{E_{i,a_{i}} T_i}, \overline{E_{j,a_{j}} T_j} ) : \forall i, \forall j \in \{ 1, \ldots , n\} , i \ne j)$). When an electrode conflict is detected ${\mathbf {V}}_{p}(n)$ is updated as described in the section “Dynamic programming for determining potential combinations”. Once $\hat{V}_{{\mathrm{min}}}(n)$ has no conflicts, $n \longleftarrow n+1$ and the algorithm continues until $V_{{\mathrm{min}}}(N)$ is found.

#### Dynamic programming for determining potential combinations

For each target $T_i$, trajectories are ranked so $v_{i,1}$ is the best trajectory (in the section “Trajectory ranking”). Initially, ${\mathbf {V}}_{p} (n) \longleftarrow [V_1]$ where $V_1 = \lbrace v_{1,1} \rbrace$ which corresponds to adding a potential plan containing the best trajectory for the $1{\mathrm{st}}$ electrode. At the next step, $n=2$, ${\mathbf {V}}_{p}(n) \longleftarrow [ \lbrace V_{{\mathrm{min}}}(n-1), v_{n,1}\rbrace ]$, which corresponds to adding the best trajectory for the $2{\mathrm{nd}}$ electrode. Algorithm 1 proceeds in this manner until electrode conflict is detected.

If $\hat{V}_{{\mathrm{min}}}(n)= \lbrace v_{1,a_{1}}, \ldots , v_{n,a_{n}} \rbrace$ has an electrode conflict ${\mathbf {V}}_{p} (n)$ is updated by:

1. Finding trajectories *i* and *j* that violate $D(\overline{E_{i,a_{i}} T_i}, \overline{E_{j,a_{j}} T_j} ) < d_{{\mathrm{traj}}}$. Note that $i \in \{1,\ldots , n-1\}$ and $j=n$ otherwise an electrode conflict would have been detected earlier.
2. Updating the $i^{\mathrm{th}}$ trajectory $\tilde{V}_{i} (n) = \min ({\mathbf {V}}_{p}(i))$.
3. Updating the $n^{\mathrm{th}}$ trajectory: $\tilde{V}_{n} (n) = \lbrace v_{1,a_{1}},\ldots , v_{i,a_{i}}, \ldots , v_{n,a_{n}+1}\rbrace$.
4. Adding $\tilde{V}_{i}(n)$ and $\tilde{V}_{n}(n)$ to ${\mathbf {V}}_{p}(n)$.

Algorithm 1 continues in this manner until $V_{{\mathrm{min}}}(N)$ is found.

### Plan visualisation and assessment

**Fig. 3:**
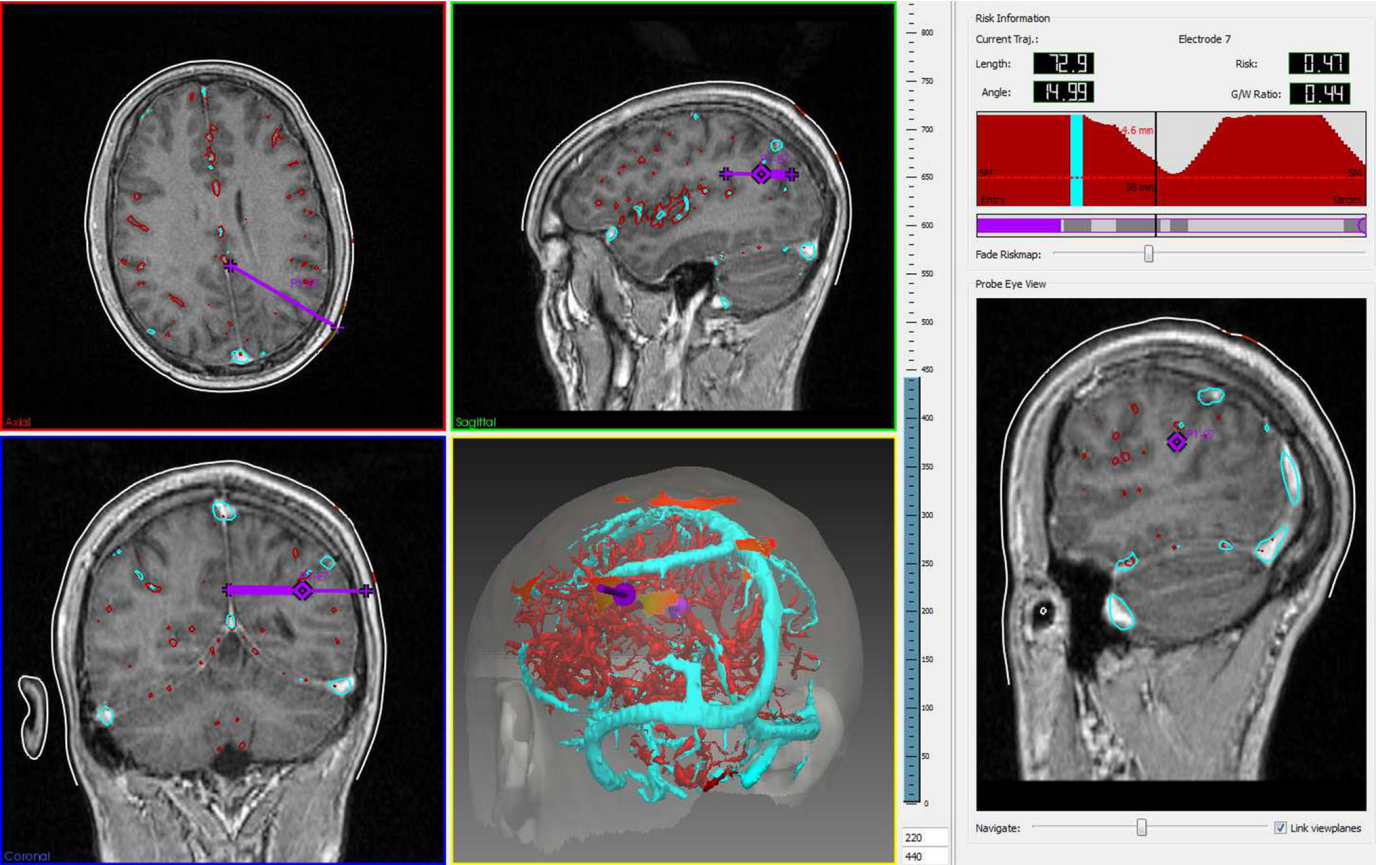
EpiNav$^{\mathrm{TM}}$ software platform. Upper panels (*left* to *right*) show the axial and sagittal imaging planes and trajectory profile described in the sections “Quantitative measures” and “Trajectory profile”. Lower panels (left to right) show the coronal imaging plane, volumetric view, and probe eye view (described in the section “Probe eye view”). The volumetric view displays critical structures (veins in *cyan*, arteries in *red*), skull template (transparent *white*), trajectory (*purple*), and entry points coloured according to risk score from low (0—*green*) to high (1—*red*). In this example, a trajectory with intermediate risk ($R_{i,a_{i}} = 0.41$—orange) is selected. The risk information shows intermediate risk is due to the trajectory traversing near an artery

Clinicians must be able to visualise and assess trajectory feasibility. We have developed the EpiNav$^{\mathrm{TM}}$ software platform to aid manual assessment with: (1) quantitative measures of trajectory suitability, (2) a trajectory profile, and (3) a probe eye view. Figure 3 displays an example layout of the EpiNav$^{\mathrm{TM}}$ software platform.

#### Quantitative measures

Four measures of trajectory suitability, length, angle, risk score, and GM-WM ratio, are displayed in the EpiNav$^{\mathrm{TM}}$ platform. Trajectory length is calculated as the length of $\overline{E T}$, where *E* is the entry point and *T* is the target. Trajectory angle is calculated as the angle of $\overline{E T}$ with respect to the skull normal. Trajectory risk score is calculated as in Equation 2, and GM-WM ratio is calculated as in Equation 3.

#### Trajectory profile

The trajectory profile provides a (a) risk profile and (b) GM profile. The risk profile displays $f_{{\mathrm{crit}}}(x)$ (described in the section “Trajectory ranking”) for $x \in \overline{E T}$. The colour of $f_{{\mathrm{crit}}}(x)$ corresponds to the closest blood vessel at *x*. The GM profile displays the colour corresponding to the tissue type (GM or WM) each electrode contact is located in. Regions of the trajectory outside the cortex are shown in the electrode colour.

Figure 3 displays the risk profile in the upper right panel. Red corresponds to trajectory regions closest to an artery, cyan to trajectory regions closest to a vein. The red line indicates the “Safety Margin” $(d_{{\mathrm{safety}}})$. The black line indicates the position of the probe eye view (Fig. 3, bottom right panel), black text shows the distance between the probe eye view and *T*, and red text shows the distance to the nearest critical structure $(f_{{\mathrm{crit}}}(x))$.

#### Probe eye view

The probe eye view displays an image perpendicular to the trajectory. This allows the user to navigate along the trajectory and assess proximity to critical structures. The probe eye view is generated by finding the geometric plane perpendicular to $x \in \overline{E T}$. Nearest neighbour interpolation calculates the intensity value for each pixel in this plane. Figure 3 displays a probe eye view in the bottom right panel.

## Experimental design and results

### Dataset description

Evaluations were performed on retrospective data from 18 patients with medically refractory epilepsy who underwent SEEG implantation. All patients had unilateral implantations with between 7 and 12 electrodes for a total of 165 electrodes. All studies involving human participants were in accordance with the ethical standards of the institutional and/or national research committee and with the 1964 Helsinki Declaration and its later amendments or comparable ethical standards. For this type of study formal consent is not required.

### Experimental design

Each electrode target was manually determined by an expert neurosurgeon relying on conventional 2*D* visualisation. Targets remained fixed across planning methods.

Parameters for STP and MTP were set as described in Table 1. These parameters were determined according to a panel of three expert neurosurgeons. $d_{{\mathrm{length}}}$, $d_{{\mathrm{angle}}}$, and $d_{{\mathrm{traj}}}$ are set according to surgically feasible approaches and available electrodes. $d_{{\mathrm{safety}}}$ is set to 3.0 mm corresponding to the minimum accuracy achievable with the neuronavigation system [18] as critical structures closer than 3.0 mm may be compromised. $d_{{\mathrm{risk}}}$ is set to 10 mm, a distance at which there is no potential to compromise critical structures. *J*, $c_j$, and $c_r$ were determined by choosing a common SEEG electrode configuration.

**Table 1:** Parameter values determined from expert neurosurgeons. $d_{{\mathrm{angle}}}$ is the largest angle safely realised with a surgical drill

Parameter	Value
$d_{{\mathrm{length}}}$	80.0 mm
$d_{{\mathrm{angle}}}$	$25^{\circ }$
$d_{{\mathrm{safety}}}$	3.0 mm
$d_{{\mathrm{risk}}}$	10.0 mm
*J*	10
$c_j$	6 mm intervals from electrode tip
$c_r$	1.2 mm
$d_{{\mathrm{traj}}}$	10.0 mm
*K*	10

$d_{{\mathrm{length}}}$ is the longest electrode. $d_{{\mathrm{safety}}}$ is the “Safety Margin”, below which trajectories have the highest risk $(R_{i,a_{i}}=1)$. $d_{{\mathrm{risk}}}$ is the “Risk Zone”, above which trajectories have no risk $(R_{i,a_{i}}=0)$. *J*, $c_j$, and $c_r$ are the number, position, and sampling radius of electrode contact points for a commonly used SEEG electrode. $d_{{\mathrm{traj}}}$ is the minimum distance for no electrode conflict. *K* is the number of histogram bins

Trajectories were assessed by length, angle, GM-WM ratio, and risk score as described in the section “Quantitative measures”. Distance to the closest sulci was also calculated in a similar fashion as distance to blood vessels described in the section “Bounding volume hierarchy (BVH) for trajectory evaluation”. These measures of trajectory suitability are biased in that STP and MTP use these measures to calculate electrode trajectories. Although the quantitative measures are biased, they assess whether a method is able to optimise multiple surgical constraints simultaneously. A qualitative analysis by an expert neurosurgeon who was blinded to the plan origin was used to complement the quantitative analysis. Qualitative analysis determined whether the implantation plans were suitable for surgery and included expert clinical knowledge that may not be captured in the quantitative measures.

**Fig. 4:**
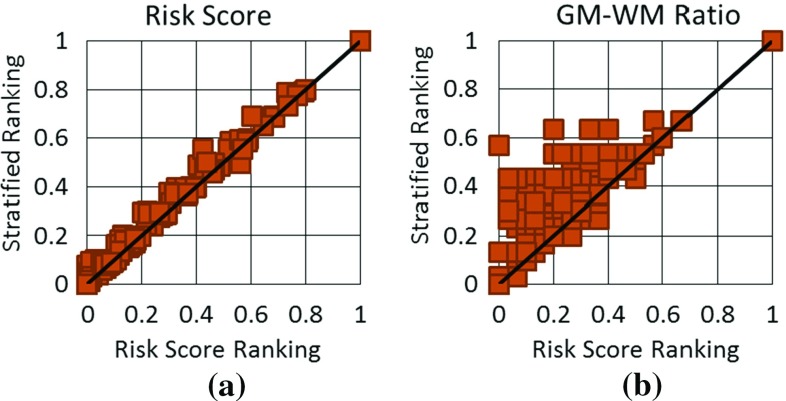
**a** Risk score and **b** GM-WM ratio for trajectories calculate by risk score ranking (plotted on the *Xaxis*) versus stratified ranking (plotted on the *Yaxis*). Points above the diagonal represent trajectories where stratified ranking increased the value compared to risk score ranking. For **a** risk score lower values (points below the diagonal) are preferred. For **b** GM-WM ratio higher values (points above the diagonal) are preferred

### Experiment 1: trajectory ranking strategies

We compared trajectories determined by risk score [29] and stratified ranking described in the section “Trajectory ranking”. Risk score ranking finds the trajectory with the lowest risk score, while stratified ranking finds a trajectory that minimises risk score while maximising GM-WM ratio. Stratified ranking is expected to increase GM-WM ratio and risk score compared to risk score ranking. A two-tailed Student’s t test was used to compare trajectories between the two ranking methods, with the null hypothesis being that the methods calculate similar trajectories.

Figure 4 displays GM-WM ratio and risk score for trajectories calculated with the two ranking methods. Both methods produced trajectories with similar risk scores, and stratified ranking increased the risk score on average $0.02 (0.0-0.13)$ and was not statistically significant $(p=0.497)$. Stratified ranking increased the GM-WM ratio by a mean of $0.08 (0.0-0.57)$ and in 22/165 trajectories by over 0.2. The difference in GM-WM ratio is statistically significant $(p=5.5 \times 10^{-7})$. Stratified ranking improved GM-WM ratio with an insignificant increase in risk score.

### Experiment 2: target order independence

We evaluated the effect of target order on MTP. For each plan, target order was randomly selected 5 times and the trajectories for each target were compared. The order targets were considered did not change the final plan, and in all cases the same trajectory was returned.

### Experiment 3: planning strategies

We compared our MTP algorithm with (a) manual planning (MP) by an expert neurosurgeon and (b) the STP algorithm described in the section “Single trajectory planning algorithm”

**Fig. 5:**
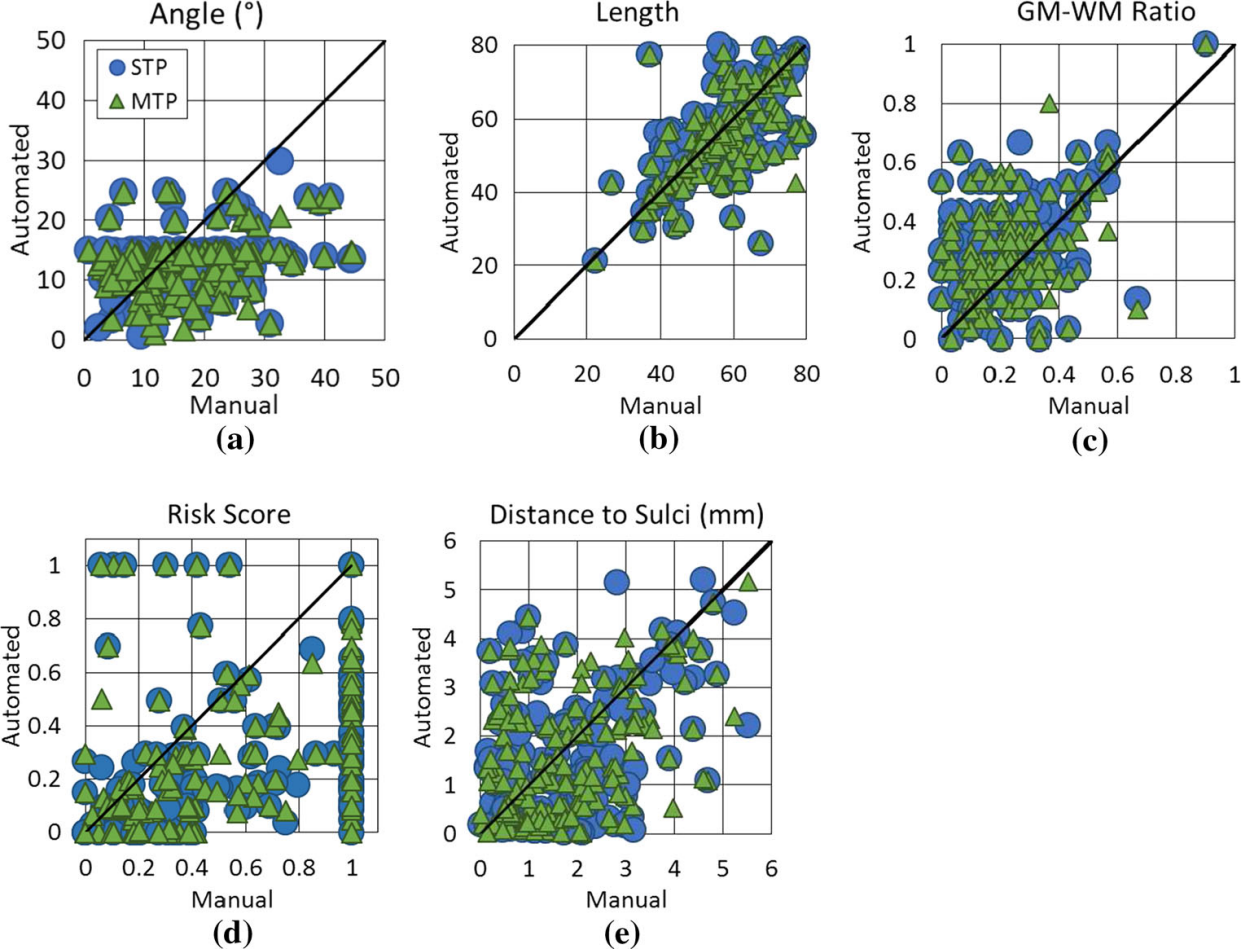
Quantitative measures for trajectories determined by MP (plotted on the *Xaxis*) versus automated planning (plotted on the *Yaxis*) for **a** angle, **b** length, **c** GM-WM ratio, **d** risk score, and **e** distance to the closest sulci. Points below the diagonal line represent trajectories where automated planning reduced the value compared to MP. For **a** angle, **b** length, and **d** risk score lower values (points below the diagonal) are preferred. For **b** GM-WM ratio and **e** distance to the closest sulci higher values (points above the diagonal) are preferred

#### Quantitative assessment

A two-tailed Student’s t test was used to compare trajectory measures between MTP and the other methods (MP, STP) with the null hypothesis being the methods return similar trajectories. To account for multiple comparisons $(n=2)$, a Bonferroni correction is applied; hence, a statistically significant value is $\alpha = 0.05/n = 0.025$.

**Fig. 6:**
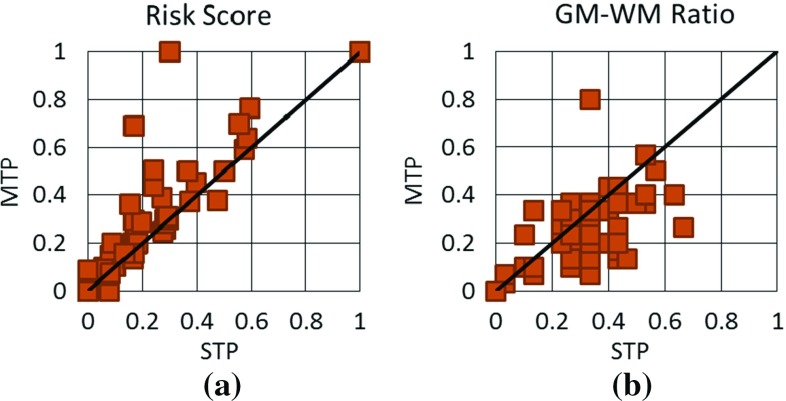
Quantitative measures of the 67 trajectories changed between STP and MTP, for **a** risk score and **b** GM-WM ratio. For **a** risk score lower values (points below the diagonal) are preferred, while for **b** GM-WM ratio higher values (points above the diagonal) are preferred

All 165 trajectories changed between MP and MTP. Figure 5 displays quantitative measures for each trajectory. MTP reduced the length of 92/165 trajectories $$(p=0.033)$$, reduced the angle with respect to the skull surface normal in 113/165 trajectories $$(p=2.7 \times 10^{-8})$$, increased GM-WM ratio in 99/165 trajectories $$(p=7.0 \times 10^{-3})$$, reduced the risk score in 122/165 trajectories $$(p=9.3 \times 10^{-8})$$, and reduced the distance to the closest sulci in 70 / 165 trajectories$$(p=0.50)$$. For 7 trajectories MTP returned trajectories with a risk score of 1, while MP returned trajectories with a risk score $$ {<}1$$; however, these MP trajectories violated the angle constraint (angle $$> d_{{\mathrm{angle}}}$$) (Fig. 6).

Between MTP and STP 69/165 trajectories, these changes were not statistically significant for any measure (*p* between 0.03 and 0.60). Although changes in quantitative measure of risk were not statistically significant, there were significant practical differences in that STP provided implantation plans that could not be surgically implemented due to electrodes being placed too close to each other. In contrast MTP found implantation plans in which each electrode trajectory was placed so that it could be implemented during surgery. MTP calculated a higher risk score than STP in 38/69 trajectories. Additionally, GM-WM ratio decreased in 42/69 trajectories. For one trajectory MTP calculated a much higher trajectory risk score (1 compared to 0.28); however, no low risk trajectories were able to avoid conflicts with other electrodes.

#### Clinical assessment

A clinical assessment of plan feasibility was performed by a single neurosurgeon, blinded to plan origin. Plans were assessed using EpiNav$^{\mathrm{TM}}$ for:

1. Avascularity: each trajectory was assessed with the probe eye view to confirm the absence of nearby blood vessels. Each plan was scored as the ratio of safe, avascular trajectories to all trajectories.
2. Conflicts: each plan was assessed in the volumetric view for conflicts between electrodes, due to either contact or inadequate spacing. Each plan was scored as the number of conflicts.
3. Feasibility: each electrode was assessed on the feasibility of surgical implementation taking into account the entry point and trajectory. Each plan was scored as the ratio of feasible electrodes to all electrodes.

**Table 2:** Measures of plan feasibility for MP, STP, and MTP obtained by one neurosurgeon blinded to plan origin

Plan	Number of electrodes	Conflicts			Avascularity			Feasibility		
		MP	STP	MTP	MP	STP	MTP	MP	STP	MTP
1	12	**0**	3	**0**	12	9	10	12	10	10
2	11	**0**	3	**0**	11	10	9	11	9	9
3	7	**0**	2	**0**	7	5	6	7	5	6
4	7	**0**	1	**0**	7	6	6	7	6	6
5	9	**0**	2	**0**	9	9	9	9	8	8
6	12	**0**	3	**0**	12	11	11	12	11	11
7	7	**0**	5	**0**	7	6	6	7	7	7
8	8	**0**	2	**0**	8	7	7	8	6	6
9	7	**0**	1	**0**	7	7	7	6	7	7
10	8	**0**	1	**0**	8	6	6	8	5	6
11	8	**0**	2	**0**	8	8	7	8	5	5
12	11	**0**	3	**0**	11	11	11	11	9	9
13	10	**0**	5	**0**	10	8	9	10	9	9
14	12	**0**	4	**0**	12	12	12	12	10	10
15	8	**0**	3	**0**	8	6	6	8	7	7
16	10	**0**	1	**0**	10	10	10	10	9	9
17	11	**0**	2	**0**	11	11	11	11	10	10
18	7	**0**	3	**0**	6	7	7	7	5	5
All	165	**0**	67	**0**	164	149	150	165	138	140

Avascularity and feasibility are reported as the ratio of electrodes that meet the criteria to all electrodes. Conflicts are reported as the number identified per plan

Bold values correspond to values where no conflicts between electrodes were found

Table 2 reports the plan feasibility measures. All three methods were effective at finding avascular trajectories for individual trajectories as determined by a neurosurgeon. This is expected as both STP and MTP are optimised according to risk score, a function of distance to critical structures. The 15 (16 for STP) trajectories that were determined to pass an unsafe distance to blood vessels were caused by the vessel segmentation algorithm not segmenting all of the small blood vessels. Only MP and MTP were effective at avoiding electrode conflicts, with the intertrajectory spacing being deemed sufficient for implementation by a neurosurgeon. Finally, all planning methods were reasonable at finding clinically feasible trajectories, although STP (138 / 165) and the MTP (140 / 165) were both inferior to MP (165 / 165). The main reasons for STP and MTP trajectories to be deemed not feasible were temporal electrodes not passing through the medial temporal lobe or orbitofrontal electrodes passing near or through the frontal sinus.

### Experiment 4: computational time

Computational efficiency of STP and MTP was evaluated. To enable a direct comparison between STP, which calculates the best trajectory for one target, and MTP, which calculates the best trajectories for *N* targets, the total time to determine all *N* trajectories was recorded. For STP the computation time is a summation of computation time for each target.

Calculations were performed on a computer with a Intel(R) Xeon(R) 12 core CPU 2.10 with 64.0 GB RAM and a single NVIDIA Quadro K4000 4 GB GPU. Table 3 reports plan computation time. All plans were computed in less than 1 min, and in a clinical setting this will enable the user to make manual adjustments to parameters and trajectories when necessary. Longer computation times were observed for plans with more electrodes (Plans 11 and 13) or electrodes that were placed in close proximity (Plan 7).

**Table 3:** Plan computation time for STP and MTP reported in seconds

Plan	Number of electrodes	Algorithm	
		STP time (sec)	MTP time (sec)
1	12	3.62	8.77
2	11	21.68	21.72
3	7	25.83	25.85
4	7	14.70	14.71
5	9	11.18	19.41
6	12	13.68	14.17
7	7	22.32	28.43
8	8	13.87	25.60
9	7	11.31	11.75
10	8	4.19	4.21
11	8	20.03	20.05
12	11	43.68	57.38
13	10	11.95	21.00
14	12	18.22	19.11
15	8	11.39	24.14
16	10	3.85	3.86
17	11	3.15	4.96
18	7	9.22	9.23
median [range]	$8.5\, [7 - 12]$	$12.77\, [3.15 - 43.68]$	$19.26\, [3.86 - 57.38]$

Plan computation time for STP is the summation of time to compute individual trajectories. The number of electrodes *N* for each plan is between 7 and 12 as indicated in the table. The final row lists the median and range of computation times for all 18 plans

**Fig. 7:**
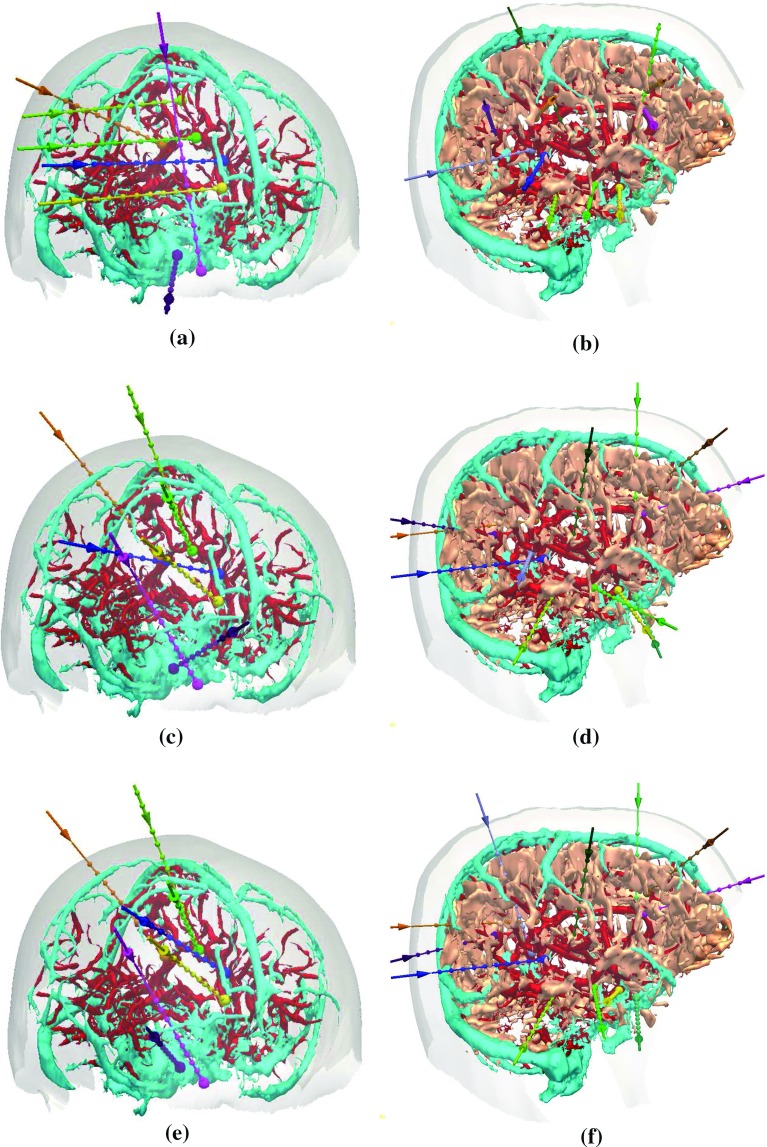
Two plans calculated by: **a**, **b** MP; **c**, **d** STP; and **e**, **f** MTP. Each plan shows the skull template (semi-transparent *white*), critical structures (arteries in *red*, veins in *cyan*, and sulci in *peach*). and trajectories [different *coloured* entry (*arrow*) and target (*sphere*)]. In **a**, **c**, and **e** sulci are not shown, so the electrode configuration can be appreciated

Computation time for the preprocessing steps was recorded. GM and cortex segmentation took ${\approx }20$ h, surface extraction for the blood vessels and sulci took between 150 and 180 s per structure, and skull segmentation and template registration took between 210 and 260 s. For SEEG electrode implantation patient scans are typically acquired at least 1 week prior to implantation planning; hence, preprocessing steps are not as time sensitive as MTP.

## Discussion

Our MTP algorithm is computational efficient, using dynamic programming to consider low risk plans in conjunction with a depth-first search algorithm to find a suitable plan. All plans evaluated containing between 7 and 12 electrodes were calculated in under a minute. Our MTP algorithm resolved electrode conflicts providing more feasible plans compared to STP. Figure 7 displays two plans determined by MP, STP, and MTP. Figure 7c displays a plan in which STP had two electrode conflicts (yellow–blue and pink–purple conflict), and such conflicts prevent the plan being surgically implemented. MTP had no conflicts as shown in Figure 7e. Figure 7d, f illustrates STP and MTP for a plan where one electrode was changed to resolve one electrode conflict (yellow–purple).

Our MTP algorithm has several differences from previously reported multiple trajectory planning algorithms [5, 6, 26, 27]. In terms of target selection, [6] required the user to select a target region from which potential target points were drawn from. [27] constrained target selection to three anatomic regions, amygdala, anterior, and posterior hippocampus. Our MTP algorithm requires the user to specify the target point.

For entry point selection [6] required the user to select an entry region on the skull. [27] defined entry map priors for each anatomic target to constrain potential entry points to surgically feasible regions. Our MTP algorithm uses a generic skull template to constrain entry points making it more flexible in the types of electrode trajectories proposed compared to other multiple trajectory planning algorithms. Constraining entry points may be desirable for some electrodes, for example to sample a specific superficial gyrus; however, it may be overly restrictive and result in nonoptimal trajectories for other electrodes.

When calculating trajectories both [6] and [27] sample target and entry regions to obtain a fixed number of trajectory combinations that are then evaluated in terms of risk and electrode conflicts. In contrast, our MTP algorithm considers all possible entry points when determining the trajectories.

Critical structures used to compute the risk score $R_{i,a_{i}}$ (in the section “Critical structure extraction”) in this work were blood vessels (arteries and veins). Trajectories that intersected sulci were rejected, but sulci were not included in calculating $R_{i,a_{i}}$. When analysing MP trajectories, it was found that while blood vessels were avoided by at least 3 mm (median value 5.11 mm), sulci were often much closer (median value 1.57 mm). Based on these results it was determined that maximising distance to sulci was not as important criteria as maximising distance to blood vessels. However, the algorithm presented to compute $R_{i,a_{i}}$ is generalisable to other structures, such as sulci or ventricles, without significantly changing task complexity or expected results. Several other state-of-the-art methods have incorporated sulci avoidance by either taking into account the trajectory angle with respect to the cortex as in [6] or including sulci in the risk metric [27].

**Fig. 8:**
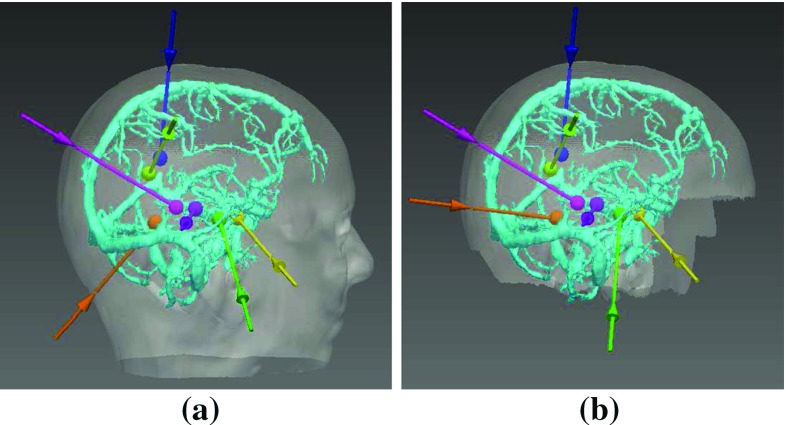
Implantation plan where potential entry points (transparent *white*) considered were the **a** patient skull or **b** skull template. Segmented critical structures were vein (*cyan*). The orange electrode trajectory has been altered, so it is above the tentorium cerebelli

The introduction of a skull template (in the section “Critical structure extraction”) allows for potential entry points to be limited to surgically feasible regions. The face, ears, and base of the skull are avoided for safety and cosmetic reasons. Figure 8 provides an example where the use of the skull template is necessary to obtain a surgically feasible plan; without the skull template an electrode (orange) would have traversed the posterior fossa inferior to the transverse sinus and penetrated the tentorium cerebelli. However, the skull template as currently implemented is limited. Due to the variability in the position and size of the ears and forehead ICP registration does not always match nonfeasible regions between the patient and template skulls. Individually tailored skull templates would reduce the number of nonfeasible entry points but would increase planning time. Even with individually tailored skull templates our MTP algorithm would still suggest some nonfeasible trajectories. This is due to certain targets having accepted trajectories that neurosurgeons are reluctant to deviate from, even when these trajectories have a higher risk score. In future work, our MTP algorithm will be modified to enable the user to restrict specific electrodes to surgically preferred regions, thereby reducing infeasible trajectories.

In a clinical setting, individual electrode trajectories that are not feasible can be manually adjusted to an appropriate trajectory. However, depending on the location of trajectories, manually adjusting one trajectory may require adjustment of other trajectories to resolve conflicts between electrodes. The EpiNav$^{\mathrm{TM}}$ software platform allows the user to fix individual electrode trajectories and rerun MTP on the remaining electrodes to find a suitable implantation plan as described in [17]. Multiple runs of MTP may be required to obtain an implantation plan in which all trajectories are safe and surgically feasible.

A thorough evaluation of our automated multiple trajectory planning algorithm for clinical use has been presented in [17]. In this study, three neurosurgeons compared 18 plans determined by MP and our MTP algorithm. All 18 plans were found to be feasible for clinical implementation. Individual trajectories were found to be safe for clinical implementation in $77.1\,\% (128/166)$ of electrodes. $10\,\% (18/166)$ of trajectories were found to be unsafe due to incorrect critical structure segmentation. To improve this performance, a more accurate vessel extraction algorithm is necessary to find avascular trajectories. $7\,\% (12/166)$ of trajectories were deemed unsafe due to proximity to sulci or the midline, highlighting the need to incorporate sulci for a clinically realistic MTP algorithm.

Stratified ranking allows for a low risk score to be prioritised with GM-WM ratio taken into consideration, provided the risk score does not substantially increase. This additional constraint is important as the goal of electrode implantation is to record EEG signals from GM, which is the site of seizure generation. Currently, the GM-WM ratio is calculated for specific contact points on the electrode for a single-electrode configuration. However, there are over a dozen different configuration of contacts on SEEG electrodes that may be implanted. Future work will include specifying the contact configuration for specific electrodes.

## Concluding remarks

We present an automated multiple trajectory planning (MTP) algorithm using depth-first searching with dynamic programming. Our algorithm was evaluated with 18 plans with between 7 and 12 electrodes. Calculation of an implantation plan took on average $19.26 (3.86-57.38)$ s. Implantation plans had a lower risk for 122 / 165 electrodes and higher grey matter-white matter (GM-WM) ratio for 99 / 165 electrodes. The computational efficiency of our algorithm enables near real-time planning of electrode implantations.

In this manuscript we focused on the development of our MTP algorithm leveraging existing methods for extracting the skull template, critical structures, and GM. Our algorithm was integrated into the EpiNav$^{\mathrm{TM}}$ software platform to enable manual assessment of calculated trajectories. A larger, prospective, comprehensive clinical study of EpiNav$^{\mathrm{TM}}$ is necessary to evaluate the utility of the software in planning intracerebral electrode implantations.
